# CLK-2/TEL2 is a conserved component of the nonsense-mediated mRNA decay pathway

**DOI:** 10.1371/journal.pone.0244505

**Published:** 2021-01-14

**Authors:** Yanwu Guo, Cristina Tocchini, Rafal Ciosk

**Affiliations:** 1 Department of Biosciences, University of Oslo, Oslo, Norway; 2 Biozentrum, University of Basel, Basel, Switzerland; 3 Institute of Bioorganic Chemistry, Polish Academy of Sciences, Poznań, Poland; East Carolina University, UNITED STATES

## Abstract

Nonsense-mediated mRNA decay (NMD) controls eukaryotic mRNA quality, inducing the degradation of faulty transcripts. Key players in the NMD pathway were originally identified, through genetics, in *Caenorhabditis elegans* as *smg* (suppressor with morphological effect on genitalia) genes. Using forward genetics and fluorescence-based NMD reporters, we reexamined the genetic landscape underlying NMD. Employing a novel strategy for mapping sterile mutations, Het-Map, we identified *clk-2*, a conserved gene previously implicated in DNA damage signaling, as a player in the nematode NMD. We find that CLK-2 is expressed predominantly in the germline, highlighting the importance of auxiliary factors in tissue-specific mRNA decay. Importantly, the human counterpart of CLK-2/TEL2, TELO2, has been also implicated in the NMD, suggesting a conserved role of CLK-2/TEL2 proteins in mRNA surveillance. Recently, variants of TELO2 have been linked to an intellectual disability disorder, the You-Hoover-Fong syndrome, which could be related to its function in the NMD.

## Introduction

RNA-controlling mechanisms are important, among others, for removing aberrant transcripts, repressing viral RNAs, and regulating gene expression [1,2]. Nonsense-mediated mRNA decay is one of the best characterized, and evolutionarily conserved, surveillance mechanisms, which, by degrading mRNAs carrying premature termination codons (PTCs), prevents synthesis of truncated proteins [2]. The NMD pathway monitors mRNA quality in cooperation with the translation machinery. During the first round of translation, exon-exon junction complexes (EJCs), deposited on mRNA during splicing, are removed from mRNA by elongating ribosomes. However, EJCs located “downstream” from PTCs, which remain associated with mRNA, facilitate the recruitment of a protein kinase, SMG1, which phosphorylates and activates a key NMD factor, UPF1. Activated UPF1 promotes the formation of a decay-inducing complex, eventually leading to the degradation of aberrant mRNA [2,3].

In addition to targeting aberrant transcripts, which arise from mutations or transcription errors, NMD regulates the expression of many “normal” mRNAs [4], and functional studies have implicated NMD in cellular differentiation, neural development, and stress responses [5–7]. The degradation of those transcripts often involves PTCs that emerge as the consequence of alternative splicing or through the engagement of upstream open reading frames (uORFs) [8,9]. Moreover, studies from various organisms indicate that a long 3’UTR is a common feature of many NMD targets [10,11]. Why long 3’UTRs render messages sensitive to NMD is not fully understood, and may involve diverse mechanisms [11,12]. At least some messages with long 3’UTRs appear to be protected from NMD by the association with PABPC1, which is important for many aspects of mRNA processing [13,14].

Genetic screens, performed in model organisms, identified many components of the NMD pathway. Seven of those, called *smg-1* to *-7* (*smg*; suppressor with morphological effect on genitalia), were originally identified in *C*. *elegans*, through random mutagenesis, as genes required to prevent expression of a message carrying PTCs [15–17]. Additional components of the nematode NMD were identified in later studies [18,19]. These findings were accompanied by similar discoveries in other models, for example the identification of *UPF1* to *-3* (corresponding to nematode *smg-2*, *smg-3*, and *smg-4*) in budding yeast [20]. Here, we employed an unbiased genetic screen to reexamine the *C*. *elegans* NMD pathway. In addition to known pathway components, our screen uncovered a novel player, CLK-2. CLK-2 (CLocK–biological timing–abnormality) impacts multiple cellular and developmental processes [21–23]. Its orthologs, from yeast to human, regulate telomere length and DNA damage response [24–27]. Additionally, biochemical studies demonstrated that the human counterpart of CLK-2, TELO2, functions as a scaffold for the assembly of a protein complex, the R2TP complex, which mediates the assembly of phosphatidylinositol 3-kinase-related kinases (PIKKs), such as ATM and ATR (critical for DNA damage response) and, importantly, the NMD kinase SMG1 [28,29]. Our finding, that CLK-2 functions as a key player in the nematode NMD, suggests that CLK-2/TEL2 proteins are evolutionarily conserved components of this surveillance pathway.

## Results

### Monitoring NMD induced by 3’UTR aberrations

**Fig 1:**
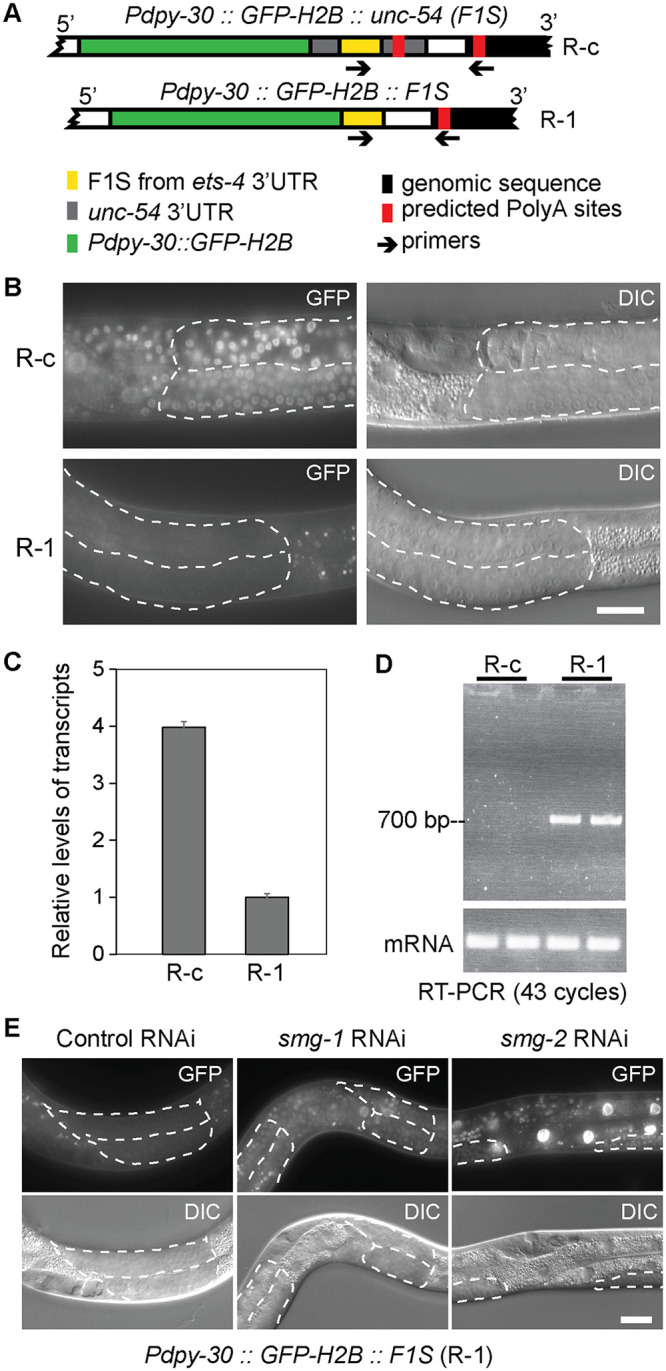
Construction of a GFP reporter strain, R-1, for the detection of NMD. **(A)** Schematics showing reporter constructs integrated into the *C*. *elegans* genome, expressing green fluorescent protein (GFP; green) fused to the histone H2B. Its expression is driven from a ubiquitous promoter, *dpy-30 (Pdpy-30)*, under the control of different 3’UTRs. A 115 bp-long fragment of *est-4* 3’UTR (F1S; yellow) was inserted either into the *unc-54* 3’UTR (upper drawing; reporter R-c), or directly downstream from the GFP-H2B open reading frame (lower drawing; reporter R-1). Arrows indicate primers, one matching the F1S sequence and the other matching genomic sequence (black) downstream from the 3’ end of the integrated construct, which were used to amplify the intervening 3’ UTR fragment. Predicted PolyA sites are indicated in red. **(B)** Partial view of animals carrying the R-c or R-1 reporters. Fluorescence micrographs are on the left, differential interference contrast (DIC) micrographs on the right. Scale bar = 20 μm. Note that the R-1 reporter is not expressed in wild-type animals. Germline tissue is highlighted by dotted lines. **(C)** RT-qPCR analysis, comparing the levels of reporter mRNAs expressed in wild-type animals. The levels of R-1 mRNA were much lower, compared to R-c mRNA. Error bars represent standard deviation. **(D)** RT-PCR, using primers indicated in A, demonstrating that the R-1 reporter yields transcripts with long 3’ UTRs, at least 700 nucleotide-long. Note that this analysis is non-quantitative. **(E)** Partial view of animals carrying the R-1 reporter. Note that the RNAi-mediated depletion of *smg-1* or *smg-2* results in de-repression of the reporter. Germline tissue is highlighted by dotted lines.

The 3’ untranslated regions (3’UTRs) contain various RNA elements that, by recruiting diverse factors, affect mRNA fate. While studying 3’UTR elements mediating degradation by a particular endonuclease, REGE-1, we created reporter strains (using Mos1-mediated Single Copy Insertion, MosSCI [30]), wherein expression of GFP-histone H2B fusion protein is controlled by 3’UTR variants (H2B concentrates GFP fluorescence to the nuclei, facilitating visualization). These variants derived from the 3’UTR of *ets-4*, a key target of REGE-1 [31]. We observed a severely diminished GFP fluorescence in one of the reporter strains, and named the corresponding reporter as “R-1” (reporter 1). In R-1, a short fragment of *ets-4* 3’UTR (called F1S, see [31]) was inserted directly after the GFP-H2B ORF stop codon (Fig 1A). The reduced GFP fluorescence correlated with reduced levels of the corresponding R-1 mRNA (Fig 1B and 1C). We hypothesized that the short 3’UTR fragment, used in the R-1 reporter, may lack signals necessary for efficient pre-mRNA processing, thus giving rise to abnormal transcripts subjected to mRNA surveillance. Using primers matching the genomic 3’ integration site of the R-1 construct, we detected an unusually long transcript, with at least 700 nucleotide-long 3’UTR (Fig 1D), which could be targeted by NMD. Indeed, we observed that RNAi-mediated depletion of NMD players, *smg-1* or *smg-2*, allowed efficient expression of the R-1 GFP (Fig 1E). Subsequent examination of the transcriptome revealed that transcripts derived from the R-1 construct contain long 3’UTRs, which appear to undergo alternative splicing (S1 Fig), possibly rendering them NMD targets.

### Identifying CLK-2 as a player in mRNA surveillance

**Fig 2:**
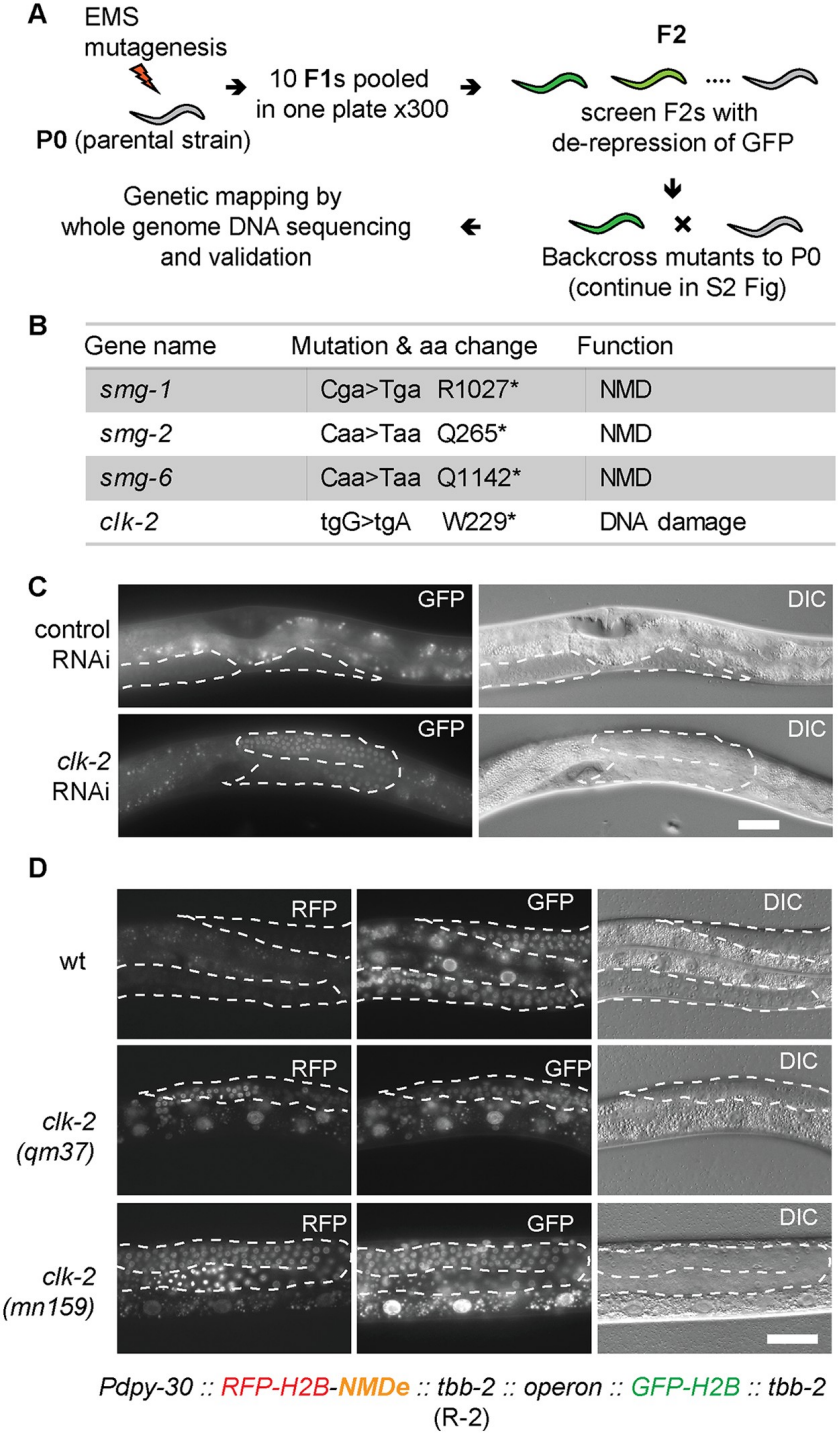
Identification of CLK-2 among NMD players, through forward genetics. **(A)** Workflow of a genetic screen for de-repression of the R-1 reporter. “P0” = parental generation; “F1, F2” = subsequent generations of the offspring. See Methods for details. **(B)** Summary of mutations identified in A. Capital letters in “Mutation” indicate mutated residues. Asterisks indicate stop codons. **(C)** Partial view of animals, carrying the R-1 reporter, subjected to *clk-2* RNAi. Scale bar = 20 μm. Germline tissue is highlighted by dotted lines. The signal in control animal comes from background gut autofluorescence. **(D)** Micrographs show partial animals, of the indicated genotypes, carrying the R-2 reporter. Scale bar = 20 μm. *Pdpy-30* is a ubiquitous promoter from the *dpy-30* gene; NMDe = NMD element. The H2B-RFP and H2B-GFP are expressed from a single operon (for details see supplementary S1 File). In wild type, the mRNA encoding H2B-RFP is degraded, due to the NMDe element, while the H2B-GFP-encoding mRNA (not subjected to NMD) is expressed. Note that, in the *clk-2* mutants, the H2B-RFP becomes expressed. Germline tissue is highlighted by dotted lines.

The NMD pathway has been extensively studied. Nevertheless, since GFP expression can be easily monitored in *C*. *elegans*, we decided to examine whether additional players in the NMD pathway could be uncovered using forward genetics and the above-described R-1 reporter. We used EMS mutagenesis to generate mutants; the work flow is illustrated in Fig 2A (for details see the Methods and S2 Fig). Conventional mutant-mapping strategies involve several backcrosses to remove DNA polymorphisms irrelevant to the mutant phenotype [32]. By contrast, we employed only one backcross, reducing necessary work and time substantially (S2 Fig, see Methods for additional information) [33]. Several mutants, displaying strong GFP expression, were recovered from this screen. The mutants were backcrossed to the parental strain once, and circa 30 independent F3 populations were collected and pooled for DNA extraction (S2 Fig). Following whole-genome sequencing and genetic mapping, we found that several mutations mapped to the *smg* genes (*smg-1*, *-2*, and *-6*), encoding known players in the NMD (Fig 2B and S3 Fig). Since a number of known NMD factors were not identified in this screen, we presume the screen was not saturated, i.e. additional components could be still identified through this approach.

Nonetheless, apart from the *smg* mutants, we isolated one mutant with a weaker GFP expression (S4 Fig). This mutant was sterile, so, to map the corresponding genetic deficiency, we modified the mapping pipeline. Instead of using homozygous F3 mutants, the heterozygous progeny of F3 animals were pooled and subjected to genome sequencing (S2B Fig). To map the underlying mutations, we employed a novel mapping strategy, which is based on the density of heterozygous sequence variants introduced by EMS (S3 Fig; for detailed explanation see the Methods). This strategy could be used, in principle, for mapping sterile mutants originating from other screens and organisms. Using this strategy, the relevant mutation was identified as a nonsense mutation in the *clk-2* gene (Fig 2B). By contrast to the *smg* mutants, in which the R-1 reporter was expressed predominantly in the soma, the *clk-2* mutation resulted in mostly gonadal expression (S4 Fig), consistent with the observed sterility. Subsequently, the gonadal NMD defect was confirmed by RNAi-mediated depletion of *clk-2* (Fig 2C). The *clk-2* sterile mutants can be maintained as heterozygotes with the *hT2* genetic balancer, containing a wild-type copy of the *clk-2* gene [34].

### Confirming CLK-2 is a bona fide player in the NMD

The efficiency of NMD is target-dependent [35]. Thus, in addition to the R-1 reporter, we designed another reporter, R-2, which was inserted (by MosSCI [30]) as a single-copy transgene for ubiquitous expression in most cell types. This reporter consists of mCherry fused to the histone H2B. Following the stop codon of mCherry::H2B ORF, the construct contains a 476 nucleotide-long fragment of the *lin-41* gene (which we called NMD element, NMDe; [36]), followed by a 3’UTR from the ubiquitously expressed *tbb-2* (tubulin) gene. The NMDe consists of the last two exons and their intervening intron. Thus, following splicing, the stop codon of the mCherry::H2B ORF in the R-2 mRNA, is recognized as a PTC, triggering NMD (for additional information on R-2 see the supplemental S1 File). Thus, in the context of a functional NMD, the R-2 reporter produces no mCherry fluorescence. By contrast, in the absence of functional NMD, the reporter mRNA escapes degradation, allowing the nuclear accumulation of mCherry-H2B fusion protein (Fig 2D). In order to rapidly distinguish between the silencing of this reporter by NMD, versus a general suppression of transcription and/or translation, the R-2 reporter was engineered to be expressed as part of an operon, which additionally encodes a second, unregulated reporter (GFP-H2B), whose expression was used as a reference (Fig 2D, S5 Fig and S1 File).

**Fig 3:**
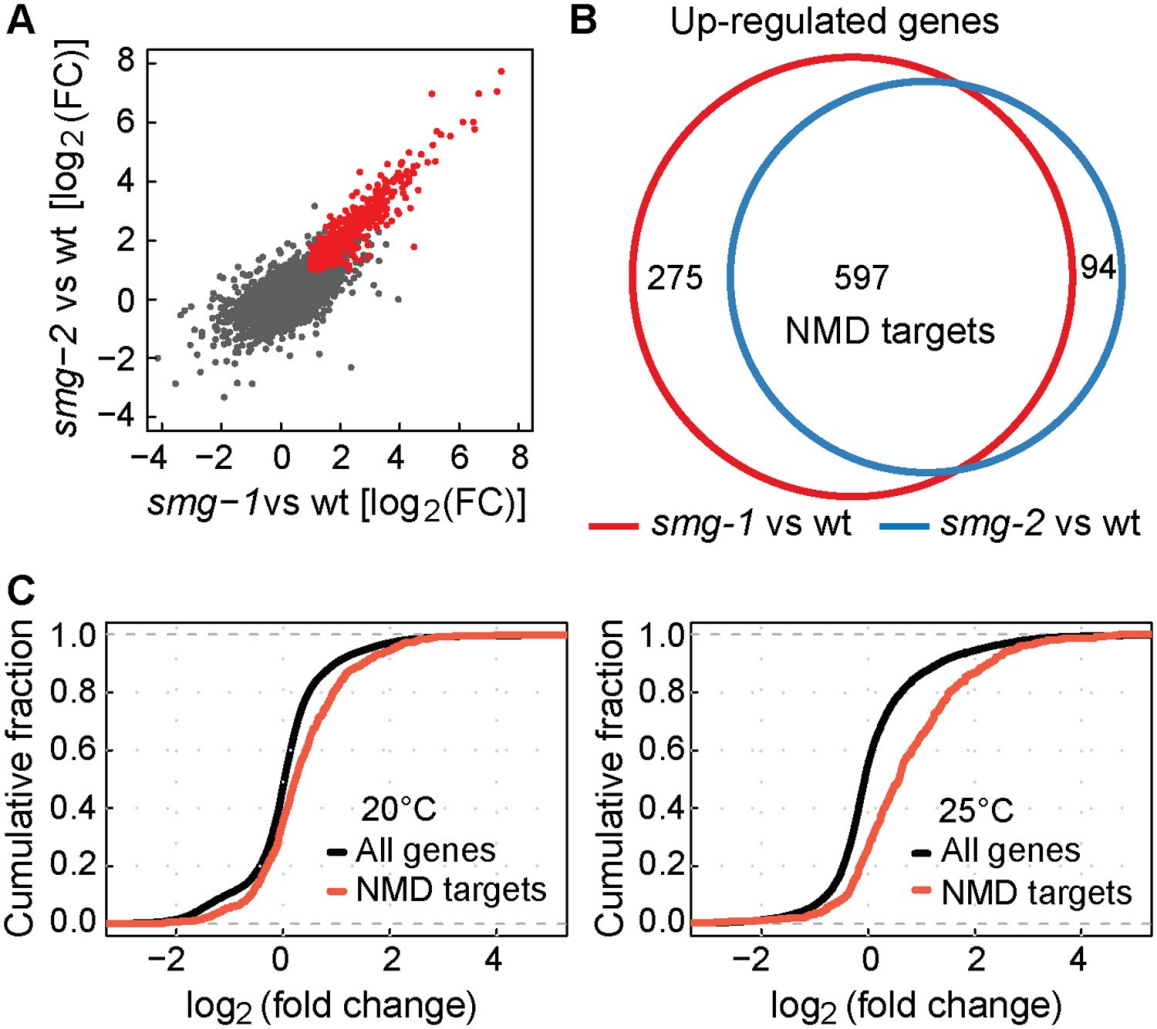
Genome-wide up-regulation of NMD mRNA targets in the absence of CLK-2. **(A)** Examination of changes in transcript levels, relative to wild type, between *smg-1(rrr59)* and *smg-2(rrr60)* mutants; correlation coefficient (*r*) of 0.838 indicates a strong correlation. In red are transcripts up-regulated at least two fold in both mutants (log2FC > 1, p-value < 0.05). “FC” = fold change. **(B)** Venn diagram highlighting the overlap between transcripts up-regulated in *smg-1*(*rrr59*) or *smg-2*(*rrr60*) mutants. The “NMD targets” are transcripts up-regulated in both mutants. **(C)** Cumulative distribution function (CDF) plots illustrating changes in the abundance of NMD targets (defined in B) in *clk-2(qm37)* mutants. Left: The animals were grown at 20°C (permissive temperature for *clk-2(qm37)*); note a modest up-regulation of NMD targets (Kolmogorov-Smirnov test, p-value < 0.0001). Right: The animals were grown at 25°C (restrictive temp.); note a stronger up-regulation of NMD targets (Kolmogorov-Smirnov test, p-value < 0.0001).

The R-2 reporter was initially validated by knocking-down known NMD factors (*smg-1*, *smg-2* and *smg-5*), whose RNAi-mediated depletion resulted in de-repression of the reporter (S5 Fig). To examine the effect of CLK-2 inhibition, we crossed the R-2 reporter to existing *clk-2*^*ts*^ (temperature sensitive) mutants; *clk-2(qm37)* and *clk-2(mn125*) [22]. We observed that the reporter became de-repressed at the restrictive (25°C) temperature (Fig 2D), suggesting a more general role for CLK-2 in the NMD. Then, to establish the extend of CLK-2 involvement in the NMD, we compared changes in the transcriptomes of *smg* and *clk-2* mutants. Initially, to determine mRNAs affected by the NMD, we examined (by RNA sequencing) *smg-1* and *smg-2* mutants, and observed a strong correlation between the changes in their transcriptomes (Fig 3A), consistent with shared functions. We arbitrarily selected genes up-regulated in both mutants as “NMD targets” (Fig 3B), though, presumably, many of these genes change in abundance due to indirect effects. Then, we examined these NMD targets in *clk-2*^*ts*^ mutants, and observed that they were up-regulated, particularly at the restricted temperature (Fig 3C). Thus, transcripts, whose abundance is normally reduced by SMG proteins, tend to be also reduced by CLK-2, suggesting that CLK-2 functions as a genuine player in the NMD pathway.

### Genetic evidence for the involvement of *C*. *elegans* R2TP complex in the NMD

**Fig 4:**
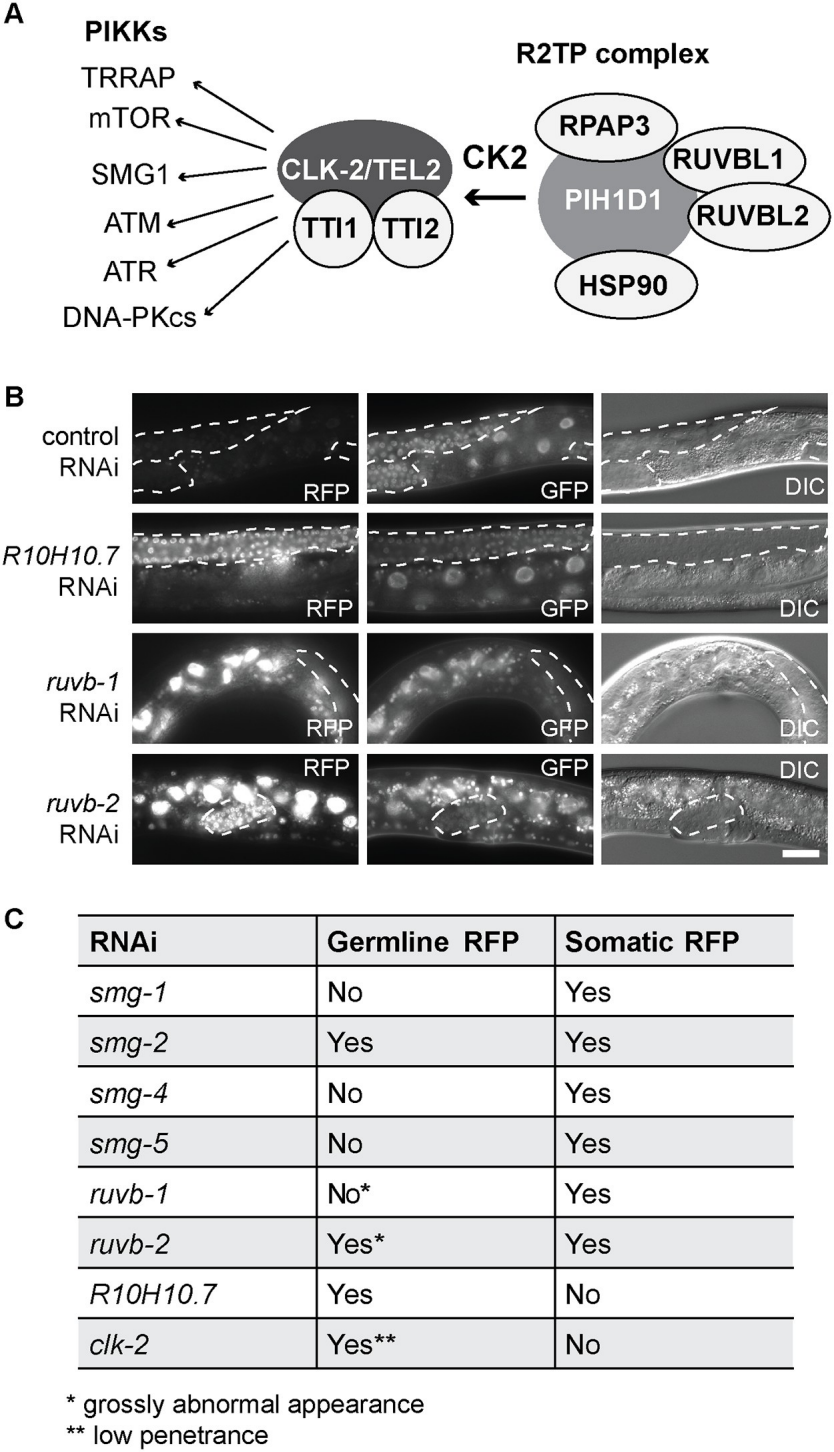
CLK-2 related factors affect the expression of NMD reporter. **(A)** Mammalian components of the R2TP complex and targeted PIKKs. Putative *C*. *elegans* counterparts are in parentheses: CK2 (*kin-3* or *kin-10*), TEL2 (*clk-2*), RPAP3 (*rpap-3*), TTI1 (*R10H10*.*7*), TTI2 (*C28H8*.*3*, with a weak similarity), SMG1 (*smg-1*), HSP90 (*hsp-90*), RUVBL1 (*ruvb-1*), and RUVBL2 (*ruvb-2*). **(B)** Partial view of animals, carrying the R-2 reporter, subjected to RNAi as indicated. Scale bar = 20 μm. Germline tissue is highlighted by dotted lines. **(C)** Summary of NMD defects, upon RNAi-mediated depletion of indicated factors, examined in animals carrying the R-2 reporter.

The human orthologue of CLK-2, TELO2, together with its interaction partners, TTI1 and TTI2 (TELO2 interacting proteins 1 or 2), coordinates the function of so-called R2TP complex [28,37]. This complex is required for the assembly of phosphatidylinositol 3-kinase-related kinases, including SMG1 (Fig 4A) [37]. To test whether CLK-2 may function in the NMD as part of an R2TP-like complex, we targeted, by RNAi, the *C*. *elegans* orthologues of the R2TP complex components; *rpap-3*, *kin-3*, *kin-10*, *ruvb-1*, *ruvb-2*, *R10H10*.*7*, *C28H8*.*3* and *hsp-90*, and observed de-repression of the R-2 reporter upon *ruvb-1*, *ruvb-2* and *R10H10*.*7* RNAi (Fig 4B and 4C).

Interestingly, we observed that RNAi-mediated depletion of R10H10.7/TTI1 affected expression of the R-2 reporter with a tissue-specific bias, as the reporter was de-repressed primarily in the germline. The preferred de-repression of the R-2 reporter was observed also upon *clk-2* RNAi and in the *clk-2(rrr58)* mutant (Fig 4C and S4 Fig). By contrast, RNAi depletion of several *smgs (-1*, *-4 and -5)* produced the opposite effect, i.e. de-repressed the reporter primarily in the soma (Fig 4C). Finally, RNAi-mediated depletion of *smg-2* or *ruvb-2* de-repressed the reporter in both soma and germline, though *ruvb-1* and *ruvb-2* RNAi-ed germlines were grossly deformed (Fig 4B and 4C), hinting at additional roles of these proteins in germline development. Together, these results suggest that individual NMD components might function with tissue-specific bias, which is consistent with studies in mammalian cells [37,38]. To explore this is a step further for CLK-2, we tagged the endogenous *clk-2* with GFP, and observed preferentially gonadal expression (S6 Fig). Thus, in case of CLK-2, its predominant NMD function in the germline may stem from its predominant expression in this tissue.

## Discussion

The NMD pathway in nematodes, in contrast to mammals, is non-essential [39]. Consequently, previous mutagenic screens, which identified *C*. *elegans* NMD components, were biased towards non-essential genes. Here, using a screening strategy allowing the recovery of sterile mutants, we identified CLK-2 as a novel player in the nematode NMD.

Typically, candidate mutations have been identified as DNA polymorphisms present in mutant animals but not wild type. An alternative strategy is to identify candidate mutations based on their linkage to mutant SNPs. Both approaches involve selection of homozygous mutants, which, in case of sterile mutations, can be challenging, as sterile animals are often few/difficult to collect. Additionally, preparation of DNA sequencing libraries from small numbers of animals can be problematic, involving specialized protocols [40]. Here, to speed up the identification of sterile *clk-2(rrr58)* mutation, we used a novel strategy. By employing mapping based on heterozygous SNP frequency, we avoided the isolation of homozygous mutants and the need for specialized library-preparing protocols. This strategy, which we dubbed heterozygous SNP frequency-based mapping (Het-Map), may be applied generally to the mapping of sterile mutations, also in other organisms.

The CLK2/TEL2 proteins are known to function in DNA damage response and telomere metabolism in various species [21,25–27]. The mammalian TEL2, alongside the R2TP complex, is thought to do so by promoting the assembly of phosphatidylinositol 3-kinase-related kinases, PIKKs, including ATM and ATR kinases that are critical for DNA repair [41]. Among target PIKKs is also SMG1, and TELO2 phosphorylation by casein-kinase 2 (CK2) has been shown to facilitate NMD by increasing the stability of SMG1 [28,37]. Thus, TELO2 and the R2TP complex function in NMD alongside DNA damage signaling. Here, we showed that the nematode counterpart of TELO2, CLK2, also functions in the NMD, and wondered whether a nematode R2TP-like complex may be involved. While targeting the putative components of nematode R2TP complex, we observed that RNAi against some, but not all, was disrupting NMD. Therefore, either RNAi against some targets was ineffective, or the putative complex may have different composition in nematodes versus humans. In mammalian cells, a key target of TELO2 in NMD is SMG1. Interestingly, RNAi-mediated depletion of *C*. *elegans* SMG-1 resulted in de-repression of the R-1 reporter in both soma and germline (Fig 1E), while the R-2 reporter appeared to be derepressed predominantly in the soma (S5 Fig). Thus, either the germline levels of SMG-1 remained sufficient to promote NMD of the R-2 reporter, or SMG-1 promotes NMD of some, but not all, germline transcripts. Testing these scenarios will be interesting as, if the latter were true, it would imply that CLK-2 promotes NMD through multiple targets, rather than only SMG-1.

To our knowledge, tissue-specific NMD has not been reported in nematodes so far, but some observations hint at the existence of tissue-specific differences. Firstly, apart from *smg-1*, RNAi against *smg-4* or *smg-5* derepressed the R-2 reporter in the soma but not germline (S5 Fig and Fig 4C). Secondly, while testing putative R2TP complex components, we noticed that RNAi of *ruvb-1* or *ruvb-2* derepressed the R-2 reporter in both germline and soma, while RNAi against *R10H10*.*7/TTI1* apparently affected only the germline (Fig 4B and 4C). Along these lines, we observed that endogenously expressed CLK-2, tagged with GFP, was expressed predominantly in the germline (S6 Fig), and *clk-2* RNAi derepressed the R-1 and R-2 reporters in the germline but not soma. Thus, CLK-2 appears to promote NMD mostly in the germline. Curiously, although the functional null allele of *clk-2* isolated here, *rrr58*, had a similar effect on NMD (monitored with the R-1 reporter; S4 Fig), compromising *clk-2* function with the temperature-sensitive mutations derepressed the R-2 reporter in both germline and soma (Fig 2D). Whether this seeming discrepancy manifests a genuine CLK-2 function in the soma, or stems from unusual features of temperature-sensitive CLK-2 variants, remains to be clarified, before attributing CLK-2 NMD function specifically to the germline.

Concluding, our results implicate CLK-2, and putative components of the nematode R2TP complex, in *C*. *elegans* NMD. Thus, although a more comprehensive analysis is needed, it appears that some form(s) of the R2TP complex play conserved functions in both DNA repair and NMD. Intriguingly, a functional connection has been observed between NMD and DNA repair in both nematodes and mammalian cells [42,43]. How exactly and why this happens is not yet clear, but one might envisage that coregulation of both processes, via CLK-2/TEL2 proteins, could help in coordinating, and potentially fine-tuning both processes.

Recently, mutations in the human TELO2 gene have been linked to the You-Hoover-Fong syndrome [44–46]. Surprisingly, You-Hoover-Fong’s patients lack hallmarks of a DNA repair syndrome, such as cancer and premature aging [47]. Considering that mutations in the NMD pathway are known to cause intellectual disability [48], we speculate that the You-Hoover-Fong syndrome may stem from defective NMD. If so, perhaps mutations in the *C*. *elegans clk-2* could be used as model for studying this disorder.

## Methods and materials

### *C*. *elegans* strains and growth conditions

*C*. *elegans* were maintained at 20°C on 2% NGM agar plates seeded with *E*. *coli* OP50, as described previously [49]. The permissive temperature for *clk-2*^ts^ mutants was 15°C. To inactivate CLK-2 in these mutants, animals grown at 20°C were transferred to 25°C for 3 hours. For information on mutants and transgenic strains used in this study, see Table 1.

**Table 1:** Stains used in this study.

Strain	Names	Ciosklab collection#
N2 Bristol	wt (wild type)	#342
*rrrSi429[Pdpy-30*::*gfp*::*h2b*::*f1s(ets-4); unc-119(+)]II*.	R-1 NMD reporter	#1814
*rrrSi387[Pdpy-30*::*mCherry*::*h2b*::*NMDE*::*tbb-2*::*operon*::*gfp*::*h2b*::*tbb-2; unc-119(+)]II*.	R-2 NMD reporter	#1632
*rrrSi428[Pdpy-30*::*gfp*::*h2b*::*unc-54(f1s(ets-4)); unc-119(+)]II*.	R-c: unc54(f1s) 3’UTR reporter	#1844
*smg-1(rrr59)I;rrrSi482[Pdpy-30*::*gfp*::*h2b*::*f1s(ets-4); unc-119(+)]II*.	*smg-1(rrr59)*	#2178
*smg-2(rrr60)I;rrrSi482[Pdpy-30*::*gfp*::*h2b*::*f1s(ets-4); unc-119(+)]II*.	*smg-2(rrr60)*	#2179
*smg-6(rrr61)III;rrrSi482[Pdpy-30*::*gfp*::*h2b*::*f1s(ets-4); unc-119(+)]II*.	*smg-2(rrr61)*	#2180
*clk-2(rrr58)III;rrrSi482[Pdpy-30*::*gfp*::*h2b*::*f1s(ets-4); unc-119(+)]II*.	*clk-2(rrr58)*	#2181
*clk-2(mn159);rrrSi[Pdpy-30*::*mCherry*::*h2b*::*NMDE*::*tbb-2*::*operon*::*gfp*::*h2b*::*tbb-2; unc-119(+)]II*.	*clk-2(mn159)*	#2182
*clk-2(qm37)III;rrrSi[Pdpy-30*::*mCherry*::*h2b*::*NMDE*::*tbb-2*::*operon*::*gfp*::*h2b*::*tbb-2; unc-119(+)]II*.	*clk-2(qm37)*	#2183
*clk-2(syb258[clk-2*::*gfp])*	*clk-2(syb258)*	#2143

### RNAi treatment

1 mM IPTG was added to an overnight culture of RNAi bacteria. 300 μl of bacterial suspension was plated on 100 μl/ml of Carbenicillin and 1 mM IPTG-containing agar plates. The L4440 empty vector was used as a negative RNAi control. Animals were placed on RNAi plates as L1 larvae.

### Fluorescent microscopy

Fluorescent images were acquired with an AxioImager.Z1 microscope (Zeiss) equipped MRm camera (Zeiss).

### RT-qPCR

Frozen pellets of about 6000 staged, young adults were subjected to RNA extraction with TRIzol as described previously [50]. Statistical methods used to calculate P values are indicated in figure legends. The primers used for RT-qPCR are listed in the supplementary S2 File.

### EMS treatment and screen

The mutagenic chemical, ethyl methanesulfonate (EMS), was applied to the R-1 reporter-carrying strain (*rrrSi482*), according to described procedures with some modifications [49]. To facilitate the screen, 10 F1s were pooled on a single plate and F2s from 300 pools were screened. Candidate mutants, expressing the R-1 GFP, were identified with a florescent microscope.

Then, to identify underlying mutations, the isolated mutants were backcrossed to P0. In case of viable mutants, animals descending from ~30 singled homozygous F2s were used for DNA extraction. In case of the sterile mutant, heterozygous mutants were backcrossed to P0. Then, animals descending from ~30 singled heterozygous F2s were used for DNA extraction (S2 Fig). Genomic DNA was extracted using Gentra Puregene Tissue Kit 4g (Qiagen) and submitted for whole genomic DNA sequencing with HiSeq2000.

### Genetic mapping by whole genomic DNA sequencing

FastQC was used to check the quality of raw sequence data [51]. Sequence reads were aligned to the ce10 *C*. *elegans* assembly using bwa with parameters: aln -n 0.04 -t 20, but only retaining single-hit alignments (‘‘bwa samse -n 1”) [52]. The resulting alignments were converted to BAM format, sorted and indexed using ‘‘samtools” [53]. Sequence variants were identified using GATK (version 3.0). Finally, the numbers of high quality (score > = 300) single nucleotide substitutions, absent from the parental strain, were counted in sequential windows of 1 Mb to identify regions of increased variant density. VCFTOOLs were used to filter out background SNPs [54]. For viable mutants, homozygous SNPs were plotted across chromosomes to estimate the location of mutated genes. For the sterile mutant, heterozygous SNPs were used for the analysis. SnpEff was employed to annotate the effects of SNP mutations [55]. Candidate genes were confirmed by RNAi or crossed with mutants obtained from the Caenorhabditis Genetics Center (CGC).

The goal of genetic mapping is to locate phenotype-causing SNPs in the genome. The rationale behind the mapping used for viable and sterile mutant was based on SNPs introduced randomly by EMS mutagenesis. The linkage between SNPs and mutated gene is distance-dependent. SNPs that are not linked to the phenotype-causing SNP/gene are diluted after backcrossing and phenotype selection. Only SNPs that are close to the phenotype-causing SNP/gene are enriched, which is revealed by SNP analysis. In case of viable mutants with recessive mutations, the mutants were homozygous for the phenotype-causing SNPs, so all corresponding sequencing reads displayed those SNPs. However, in case of the recessive sterile mutant, the progeny of heterozygous mutant were collected. Based on Mendelian genetics, these were ¼ wild-type, ½ heterozygous, and ¼ sterile. On average, half of the corresponding sequencing reads displayed the phenotype-causing SNP and the other half the wild-type sequence. In what we called “the heterozygous SNP frequency-based mapping”, or Het-Map for short, the location of phenotype-causing mutation was pinpointed based on the increased co-occurrence between the phenotype and “heterozygous” SNPs.

### RNAseq and data analysis

Synchronized young adult worms were collected from NG 2% plates using M9 buffer. The worm pellets were frozen in liquid nitrogen before stored at -80°C. Frozen pellets of young adult worms were subjected to RNA extraction as described previously [50]. rRNA was depleted from RNA using the Ribo-Zero Magnetic Kit (MRZ11124C, Epicenter). Subsequently, RNA was purified with RNA Cleanup & Concentrator from Zymo Research. Quality of RNA was monitored by Bioanalyzer RNA Pico chip. The library was prepared using the ScriptSeq v2 RNA-Seq Library Preparation Kit (Epicentre).

FASTQC was used to check the quaility of the raw sequence data. The reads were mapped to C. elegans genome (Ensembl WBcel235) using STAR aligner with default parameters except: outFilterMismatchNmax 3,outFilterMultimapNmax 1, alignIntronMax 15000, outFilterScoreMinOverLread 0.33, outFilterMatchNminOverLread 0.33. Count matrices were generated for the number of reads overlapping with the exons of protein coding genes using summarizeOverlaps from GenomicFeatures. Gene expression levels (exonic) from RNA-seq data were quantified as described previously. The differentially expressed genes were analyzed using DEseq2 [56].

To analyze the 3’UTR structure of the abnormal 3’UTR reporter, the sequence of the injection vector was used as a reference for alignment. STAR aligner was used for RNAseq data alignment [57]. The genomic data have been deposited at the GEO with accession number GEO: GSE156517.

## Supporting information

S1 Fig IGV browser view of RNAseq alignment to the integrated R-1 reporter. Top: Shown are elements of the integrated R-1 reporter. Below: Each horizontal bar represents one RNAseq read (colored reads indicate inferred insertion or deletion, according to the IGV browser). Breaks between the reads are indicative of splicing; putative introns are indicted as thin horizontal lines. The gene encoding histone H2B is present in the genome in multiple copies, hence the RNA track of H2B is much higher than of GFP (with 3 introns in the construct). Note that transcripts generated from the construct are apparently subjected to splicing in the 3’UTR region. Scale bar: 500 bp.
(PDF)

S2 Fig Genetic mapping strategies. **(A)** Non-essential mutations: Mutants isolated from the screen were backcrossed to the parental strain (P0, carrying the R-1 reporter). Animals of the F2 generation were singled and allowed to produce F3s. Then, genomic DNA was extracted from about 30 pooled homozygous F3 populations, and subjected to high-throughput sequencing. Candidate mutations were identified by homozygous SNPs analysis. Black: Wild-type animals; green: Homozygous mutants; gray: Heterozygous mutants. **(B)** Sterile mutation: Heterozygous mutant isolated from the screen was backcrossed to the parental strain (P0, carrying the R-1 reporter). Animals of the F2 generation were singled and allowed to produce F3s. Then, genomic DNA was extracted from about 30 pooled heterozygous F3 populations, and subjected to high-throughput sequencing. Candidate mutation was identified by heterozygous SNPs analysis.
(PDF)

S3 Fig Analysis of genomic DNA sequencing to map mutations. Plots facilitating mapping of mutations in: *smg-6(rrr61)* (**A-B**; *smg-6* is on chromosomes III.), *smg-2(rrr60)* (**C-D**; *smg-2* is on chromosomes I.), *clk-2(rrr58)* (**E-F**; *clk-2* is on chromosomes III.), and *smg-1(rrr59)* (**G-H**; *smg-1* is on chromosomes I.). Dot plots (**A, C, E, G**) show the distribution of SNPs along chromosomes; heterozygous SNPs are in red and homozygous in black. The y-axis indicates the quality of SNPs. Bar plots (**B, D, F, H**) shown the density of SNPs whose quality is above 300. The approximate location of candidate mutated genes was mapped based on these plots; a candidate mutation associates with a region bearing SNPs of high quality and density. Note that for SNPs density plot F, the heterozygous SNPs were used. For others, homozygous SNPs were used to generate the plots.
(PDF)

S4 Fig Selected NMD mutants, *clk-2* and *smg-1*, isolated following EMS mutagenesis. Partial view of animals, of the indicated genotypes, carrying the R-1 reporter. Scale bar = 20 μm. Germline tissue is highlighted by dotted lines.
(PDF)

S5 Fig Testing NMD with the R-2 reporter. Partial view of animals, carrying the R-2 reporter, subjected to RNAi as indicated. Scale bar = 20 μm. Germline tissue is highlighted by dotted lines.
(PDF)

S6 Fig CLK-2 expression in the germline. Partial view of an animal *clk-2(syb258)* expressing GFP-tagged, endogenous CLK-2. Scale bar = 20 μm. Germline tissue is highlighted by dotted lines.
(PDF)

S1 File The construct of R-2 NMD reporter. (PDF)

S2 File Primers used in the study. (XLSX)
