# Supplementary figures and images for: CLK-2/TEL2 is a conserved component of the nonsense-mediated mRNA decay pathway
Source: PLoS One. 2021 Jan 14;16(1):e0244505. doi: 10.1371/journal.pone.0244505 (PMC7808604; doi:10.1371/journal.pone.0244505)

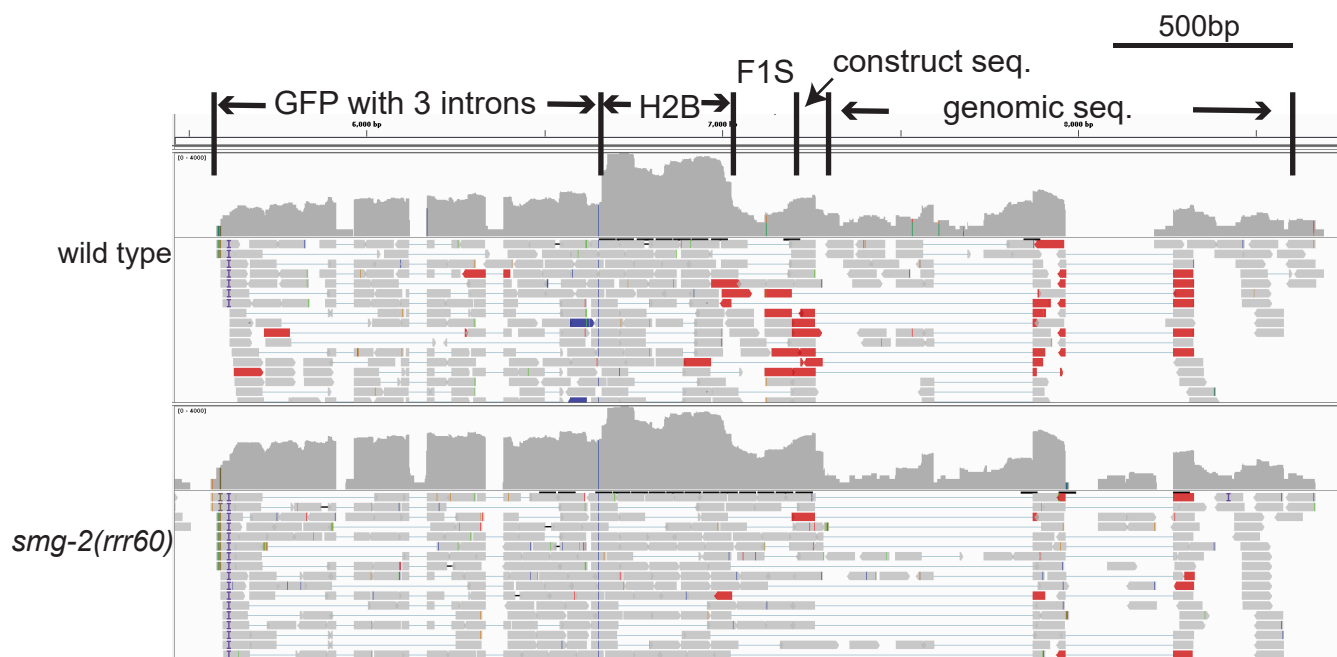

Supplement: S1 Fig — Top: Shown are elements of the integrated R-1 reporter. Below: Each horizontal bar represents one RNAseq read (colored reads indicate inferred insertion or deletion, according to the IGV browser). Breaks between the reads are indicative of splicing; putative introns are indicted as thin horizontal lines. The gene encoding histone H2B is present in the genome in multiple copies, hence the RNA track of H2B is much higher than of GFP (with 3 introns in the construct). Note that transcripts generated from the construct are apparently subjected to splicing in the 3’UTR region. Scale bar: 500 bp. (PDF) [file pone.0244505.s001.pdf]

**A**

For non-essential mutations:

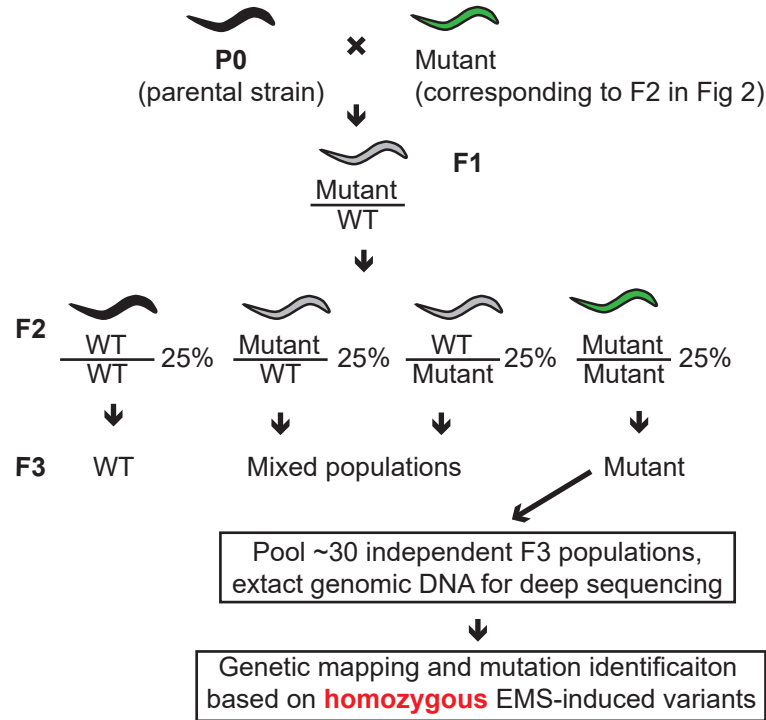**B**

For the sterile mutation:

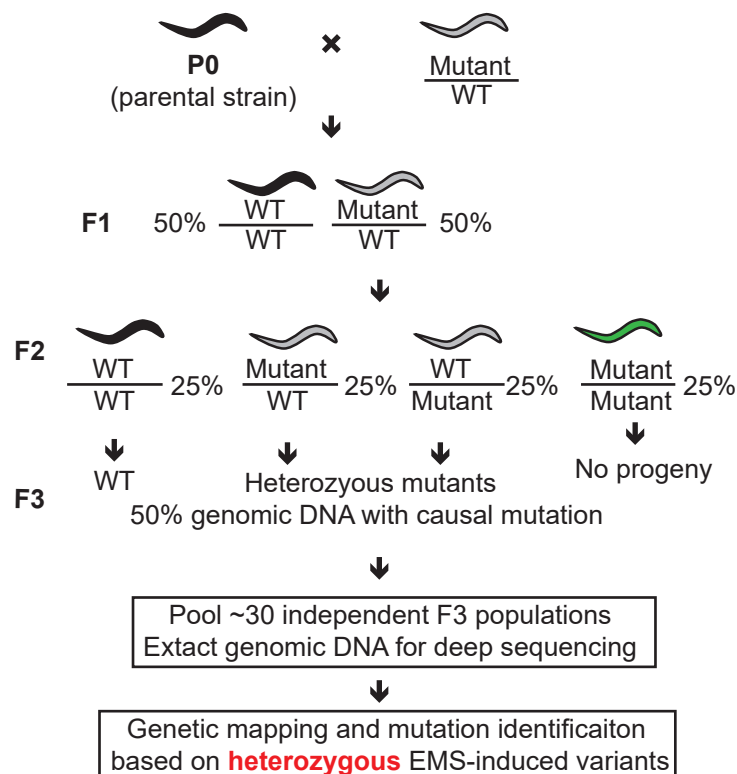

Supplement: S2 Fig — (A) Non-essential mutations: Mutants isolated from the screen were backcrossed to the parental strain (P0, carrying the R-1 reporter). Animals of the F2 generation were singled and allowed to produce F3s. Then, genomic DNA was extracted from about 30 pooled homozygous F3 populations, and subjected to high-throughput sequencing. Candidate mutations were identified by homozygous SNPs analysis. Black: Wild-type animals; green: Homozygous mutants; gray: Heterozygous mutants. (B) Sterile mutation: Heterozygous mutant isolated from the screen was backcrossed to the parental strain (P0, carrying the R-1 reporter). Animals of the F2 generation were singled and allowed to produce F3s. Then, genomic DNA was extracted from about 30 pooled heterozygous F3 populations, and subjected to high-throughput sequencing. Candidate mutation was identified by heterozygous SNPs analysis. (PDF) [file pone.0244505.s002.pdf]

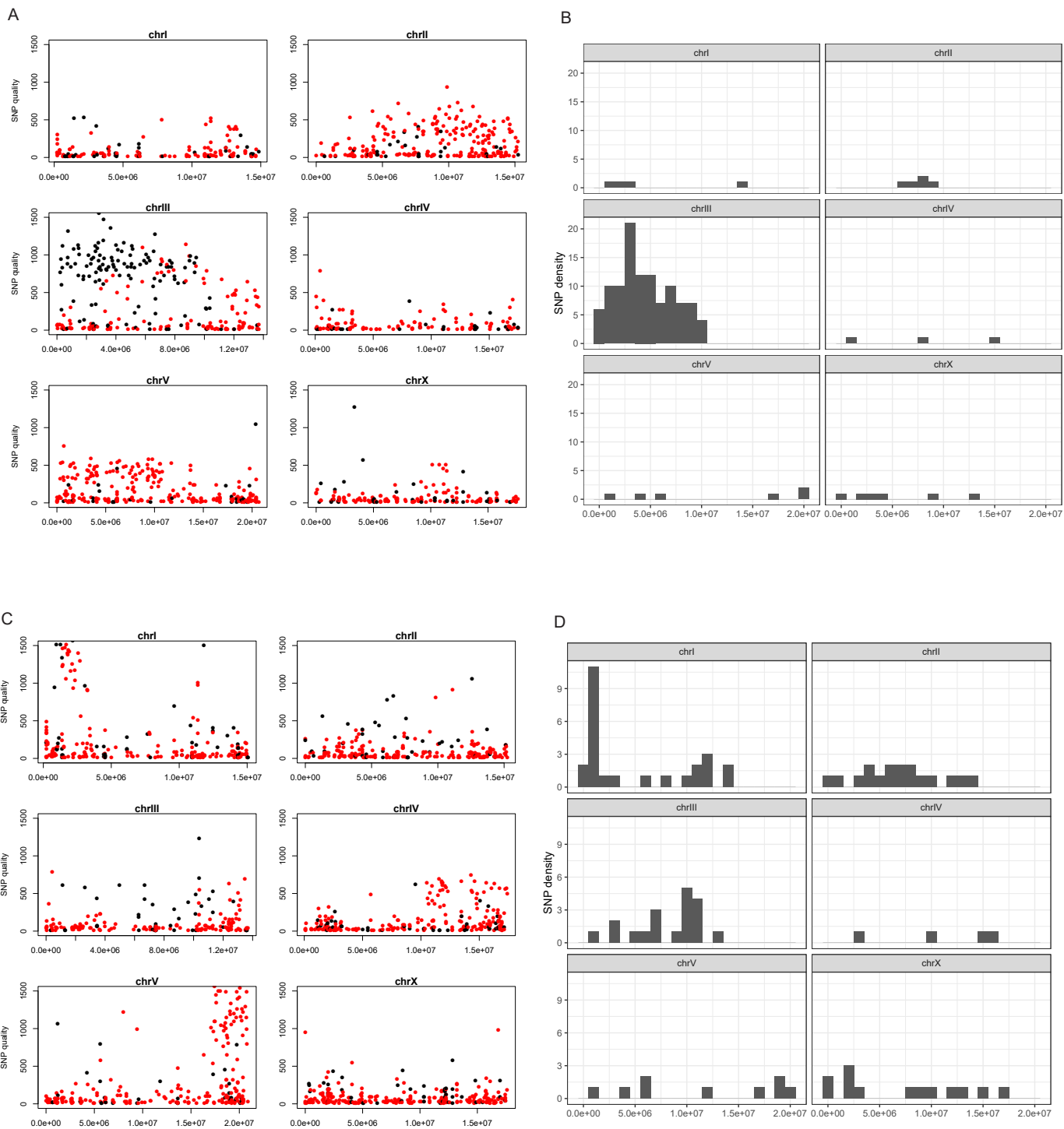

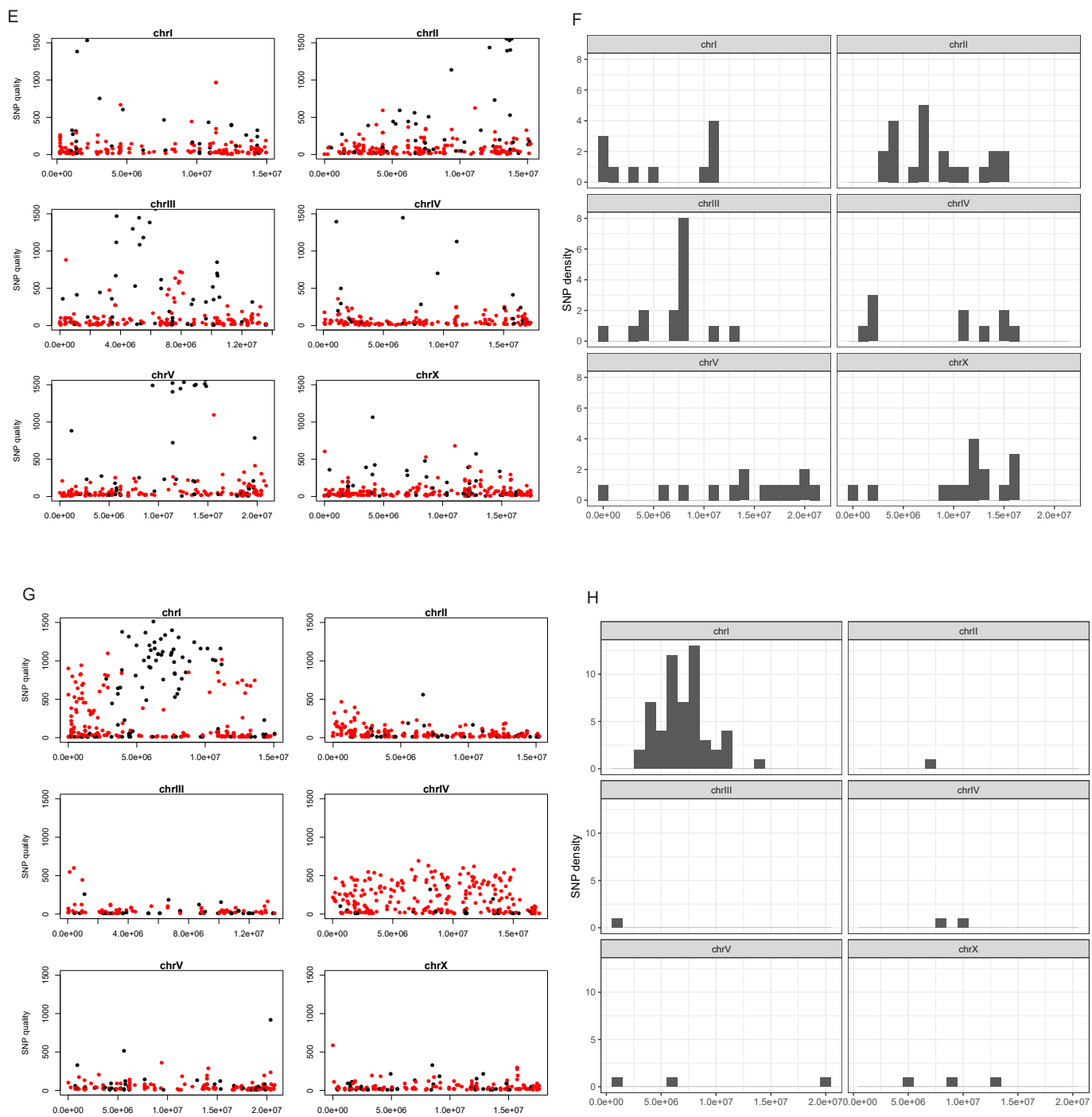

Supplement: S3 Fig — Plots facilitating mapping of mutations in: smg-6(rrr61) (A-B; smg-6 is on chromosomes III.), smg-2(rrr60) (C-D; smg-2 is on chromosomes I.), clk-2(rrr58) (E-F; clk-2 is on chromosomes III.), and smg-1(rrr59) (G-H; smg-1 is on chromosomes I.). Dot plots (A, C, E, G) show the distribution of SNPs along chromosomes; heterozygous SNPs are in red and homozygous in black. The y-axis indicates the quality of SNPs. Bar plots (B, D, F, H) shown the density of SNPs whose quality is above 300. The approximate location of candidate mutated genes was mapped based on these plots; a candidate mutation associates with a region bearing SNPs of high quality and density. Note that for SNPs density plot F, the heterozygous SNPs were used. For others, homozygous SNPs were used to generate the plots. (PDF) [file pone.0244505.s003.pdf]

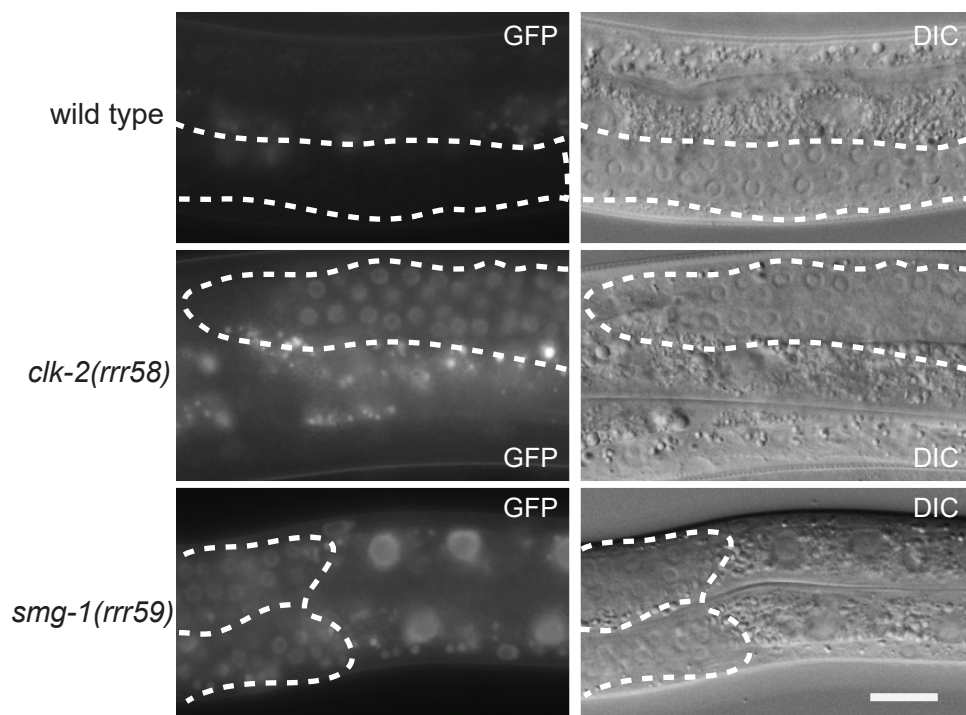

Supplement: S4 Fig — Partial view of animals, of the indicated genotypes, carrying the R-1 reporter. Scale bar = 20 μm. Germline tissue is highlighted by dotted lines. (PDF) [file pone.0244505.s004.pdf]

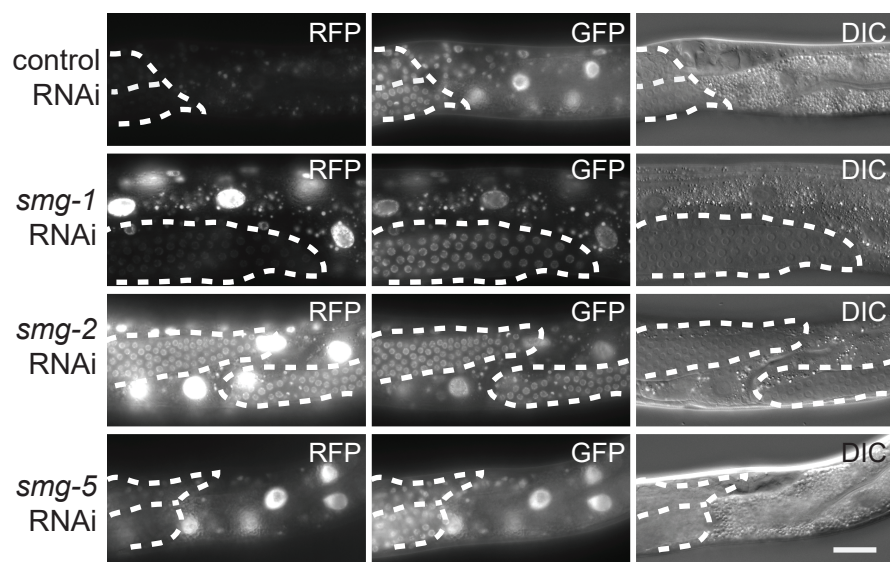

Supplement: S5 Fig — Partial view of animals, carrying the R-2 reporter, subjected to RNAi as indicated. Scale bar = 20 μm. Germline tissue is highlighted by dotted lines. (PDF) [file pone.0244505.s005.pdf]

*clk-2(syb258)*

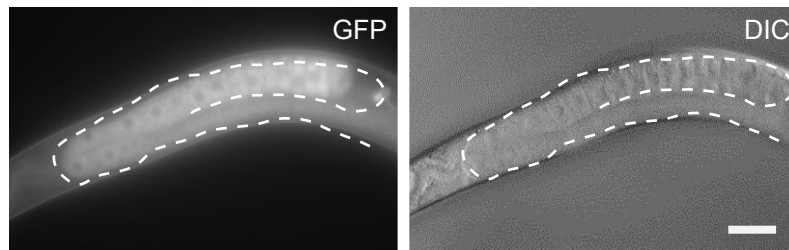

Supplement: S6 Fig — Partial view of an animal clk-2(syb258) expressing GFP-tagged, endogenous CLK-2. Scale bar = 20 μm. Germline tissue is highlighted by dotted lines. (PDF) [file pone.0244505.s006.pdf]
