# Supplementary material for: CLK-2/TEL2 is a conserved component of the nonsense-mediated mRNA decay pathway
Source: PLoS One. 2021 Jan 14;16(1):e0244505. doi: 10.1371/journal.pone.0244505 (PMC7808604; doi:10.1371/journal.pone.0244505)
Supplement: S1 File — (PDF) [file pone.0244505.s007.pdf]

Panel A

Color code for the different parts of the R-2 reporter transgene (valid for this and the following panels of this figure) followed by its unspliced sequence with untranslated and regulatory regions. Introns are shown in lowercase.

mCherry linker H2B NMDe tbb-2 3'UTR operon GFP linker H2B tbb2 3'UTR

>unspliced + UTRs_R-2 sequence

```
ATGGCAATTATTAAAGAGTTTATGCGTTTCAAGGTGCATATGGAGGGATCTGTCAATGGGCATGAGTTTGAAATTGAAGGTGAAGGAGAAGGCCGACCATATGAGGGAACACAAAC
CGCAAAACTAAAGgtaagtttaaacatatatatactaactaaccctgattatttaaattttcagGTAACTAAAGGCGGACCATTACCATTCGCCTGGGACATCCTCTCTCCACAGT
TCATGTATGGAAGTAAAGCTTATGTTAAACATCCGGCAGATATACCAGATTATTTGAAACTTTCATTCCCGGAGGGTTTTAAGTGGGAACGCGTAATGAATTTTGAAGACGGAGGA
GTTGTTACAGTGACGCAAGACTCAAGgtaagtttaaacagttcggtactaactaaccatacatatttaaattttcagCCTCCAAGATGGAGAATTTATTTATAAAGTCAAACTTCG
AGGAACGAATTTCCCCTCGGATGGACCTGTTATGCAGAAGAAGACTATGGGATGGGAAGCTTCAAGTGAAAGAATGTACCCTGAAGACGGTGCTCTTAAGGGAGAGATTAAACAAC
GTCTTAAATTGAAAGATGGAGGACATTACGATGCTGAGgtaagtttaaacatgattttactaactaactaatctgatttaaattttcagGTGAAGACAACTTACAAAGCCAAAAAA
CCAGTTCAGCTGCCAGGAGCGTACAATGTTAATATTAAACTGGATATCACCTCCCACAACGAGGATTACACTATCGTTGAGCAATATGAAAGAGCTGAAGGGCGGCACTCGACAGG
TGGCATGGATGAATTGTATAAGTGCCCGGGGGATCGGTGGAGCTCCACCGGTGGCGGCCGCTCTAGAACTAGTCCACCAAAGCCATCTGCCAAGGGAGCCAAGAAGGCCGCCAAGA
CCGTCGTTGCCAAGCCAAAGGACGGAAAGAAGAGACGTCATGCCCGCAAGGAATCGTACTCCGTCTACATCTACCGTGTTCTCAAGCAAGTTCACCCAGACACCGGAGTCTCCTCC
AAGGCCATGTCTATCATGAACTCCTTCGTCAACGATGTATTCGAACGCATCGCTTCGGAAGCTTCCCGTCTTGCTCATTACAACAAACGCTCAACGATCTCATCCCGCGAAATTCA
AACCGCTGTCCGTTTGATTCTCCCAGGAGAACTTGCCAAGCACGCCGTGTCTGAGGGAACCAAGGCCGTCACCAAGTACACTTCCAGCAAGTAACTTCAACAATCACCGACTCGCA
GTCCTATCGCCCAGAAACATGTCAGAGATGAAGGTTTACGGAAGTGAAGGAGATGGTGATGGAATGTTCGTTCGTCCACAAGGAGTTGTGATTGATCCAGAGGGACATATACTTGT
ATGTGACTCTCGGAACAATCGAGTTCAAGTCTTTGCGTCTGATGATATGAGATTCATTGGCTCATTTGGACTTGGACCCGTCCCCAATTCTGGGTTCCAAATGCCACAAGAGCTAC
CGGCTCCGTATTCGgttagtttttgatttcaagaaatgtttttgtatttgtacttttcagTCTCTCGGTGGTCCATTCGGTGCTCCAGCCTTTTCATCGGCTCCAACTCCACTGAC
TCCTTCACCACGTCAGCTTCTGGATCGTCCCACTGATTTGGCAGTTGGACCTGATGGTCGCATATACGTCGTTGATTTCGGAAACAATTGCATCCGTGTCTTCTAGATGCAAAATC
CTTTCAAGCATTCCCTTCTTCTCTATCACTCTTCTTTCTTTTTGTCAAAAAATTCTCTCGCTAATTTATTTGCTTTTTTAATGTTATTATTTTATGACTTTTTATAGTCACTGAAA
AGTTTGCATCTGAGTGAAGTGAATGCTATCAAAATGTGATTCTGTCTGATGTACTTTCACAATCTCTCTTCAATTCCATTTTGAAGTGCTTTAAACCCGAAAGGTTGAGAAAAATG
CGAGCGCTCAAATATTTGTATTGTGTTCGTTGAGTGACCCAACAAAAAGAGGAAACTTTATTGTGCCGCCAAGAAAAAAGTCTCAGACCCAGCTTTCTTGTACAAAGTGGAATAAA
GGTTGTATATTTATTCATCTTATTGAATCTGCCATTTCCTCGTTTTTGCGAGTTTATATACCTTCCAATTTTCTTTCTATTGTATTTTCAACTTCTAATTTTAATTCAGGGAAACT
GCTTCAACGCATCATGAGTAAAGGAGAAGAACTTTTCACTGGAGTTGTCCCAATTCTTGTTGAATTAGATGGTGATGTTAATGGGCACAAATTTTCTGTCAGTGGAGAGGGTGAAG
GTGATGCAACATACGGAAAACTTACCCTTAAATTTATTTGCACTACTGGAAAACTACCTGTTCCATGGgtaagtttaaacatatatatactaactaaccctgattatttaaatttt
cagCCAACACTTGTCACTACTTTCTGTTATGGTGTTCAATGCTTCTCGAGATACCCAGATCATATGAAACGGCATGACTTTTTCAAGAGTGCCATGCCCGAAGGTTATGTACAGGA
AAGAACTATATTTTTCAAAGATGACGGGAACTACAAGACACgtaagtttaaacagttcggtactaactaaccatacatatttaaattttcagGTGCTGAAGTCAAGTTTGAAGGTG
ATACCCTTGTTAATAGAATCGAGTTAAAAGGTATTGATTTTAAAGAAGATGGAAACATTCTTGGACACAAATTGGAATACAACTATAACTCACACAATGTATACATCATGGCAGAC
AAACAAAAGAATGGAATCAAAGTTgtaagtttaaacatgattttactaactaactaatctgatttaaattttcagAACTTCAAAATTAGACACAACATTGAAGATGGAAGCGTTCA
ACTAGCAGACCATTATCAACAAAATACTCCAATTGGCGATGGCCCTGTCCTTTTACCAGACAACCATTACCTGTCCACACAATCTGCCCTTTCGAAAGATCCCAACGAAAAGAGAG
ACCACATGGTCCTTCTTGAGTTTGTAACAGCTGCTGGGATTACACATGGCATGGATGAACTATACAAATGCCCGGGGGATCGGTGGAGCTCCACCGGTGGCGGCCGCTCTAGAACT
AGTCCACCAAAGCCATCTGCCAAGGGAGCCAAGAAGGCCGCCAAGACCGTCGTTGCCAAGCCAAAGGACGGAAAGAAGAGACGTCATGCCCGCAAGGAATCGTACTCCGTCTACAT
CTACCGTGTTCTCAAGCAAGTTCACCCAGACACCGGAGTCTCCTCCAAGGCCATGTCTATCATGAACTCCTTCGTCAACGATGTATTCGAACGCATCGCTTCGGAAGCTTCCCGTC
TTGCTCATTACAACAAACGCTCAACGATCTCATCCCGCGAAATTCAAACCGCTGTCCGTTTGATTCTCCCAGGAGAACTTGCCAAGCACGCCGTGTCTGAGGGAACCAAGGCCGTC
ACCAAGTACACTTCCAGCAAGTAAATGCAAAATCCTTTCAAGCATTCCCTTCTTCTCTATCACTCTTCTTTCTTTTTGTCAAAAAATTCTCTCGCTAATTTATTTGCTTTTTTAAT
GTTATTATTTTATGACTTTTTATAGTCACTGAAAAGTTTGCATCTGAGTGAAGTGAATGCTATCAAAATGTGATTCTGTCTGATGTACTTTCACAATCTCTCTTCAATTCCATTTT
GAAGTGCTTTAAACCCGAAAGGTTGAGAAAAATGCGAGCGCTCAAATATTTGTATTGTGTTCGTTGAGTGACCCAACAAAAAGAGGAAACTTTATTGTGCCGCCAAGAAAAAAGTC
TCA
```

Panel B

Spliced sequence of the NMD-sensitive region of the R-2 reporter.

```
>spliced_R-2 sequence
ATGGCAATTATTAAAGAGTTTATGCGTTTCAAGGTGCATATGGAGGGATCTGTCAATGGGCATGAGTTTGAAATTGAAGGTGAAGGAGAAGGCCGACCATATGAGGGAACACAAAC
CGCAAAACTAAAGGTAACTAAAGGCGGACCATTACCATTCGCCTGGGACATCCTCTCTCCACAGTTCATGTATGGAAGTAAAGCTTATGTTAAACATCCGGCAGATATACCAGATT
ATTTGAAACTTTCATTCCCGGAGGGTTTTAAGTGGGAACGCGTAATGAATTTTGAAGACGGAGGAGTTGTTACAGTGACGCAAGACTCAAGCCTCCAAGATGGAGAATTTATTTAT
AAAGTCAAACTTCGAGGAACGAATTTCCCCTCGGATGGACCTGTTATGCAGAAGAAGACTATGGGATGGGAAGCTTCAAGTGAAAGAATGTACCCTGAAGACGGTGCTCTTAAGGG
AGAGATTAAACAACGTCTTAAATTGAAAGATGGAGGACATTACGATGCTGAGGTGAAGACAACTTACAAAGCCAAAAAACCAGTTCAGCTGCCAGGAGCGTACAATGTTAATATTA
AACTGGATATCACCTCCCACAACGAGGATTACACTATCGTTGAGCAATATGAAAGAGCTGAAGGGCGGCACTCGACAGGTGGCATGGATGAATTGTATAAGTGCCCGGGGGATCGG
TGGAGCTCCACCGGTGGCGGCCGCTCTAGAACTAGTCCACCAAAGCCATCTGCCAAGGGAGCCAAGAAGGCCGCCAAGACCGTCGTTGCCAAGCCAAAGGACGGAAAGAAGAGACG
TCATGCCCGCAAGGAATCGTACTCCGTCTACATCTACCGTGTTCTCAAGCAAGTTCACCCAGACACCGGAGTCTCCTCCAAGGCCATGTCTATCATGAACTCCTTCGTCAACGATG
TATTCGAACGCATCGCTTCGGAAGCTTCCCGTCTTGCTCATTACAACAAACGCTCAACGATCTCATCCCGCGAAATTCAAACCGCTGTCCGTTTGATTCTCCCAGGAGAACTTGCC
AAGCACGCCGTGTCTGAGGGAACCAAGGCCGTCACCAAGTACACTTCCAGCAAGTAACTTCAACAATCACCGACTCGCAGTCCTATCGCCCAGAAACATGTCAGAGATGAAGGTTT
ACGGAAGTGAAGGAGATGGTGATGGAATGTTCGTTCGTCCACAAGGAGTTGTGATTGATCCAGAGGGACATATACTTGTATGTGACTCTCGGAACAATCGAGTTCAAGTCTTTGCG
TCTGATGATATGAGATTCATTGGCTCATTTGGACTTGGACCCGTCCCCAATTCTGGGTTCCAAATGCCACAAGAGCTACCGGCTCCGTATTCGTCTCTCGGTGGTCCATTCGGTGC
TCCAGCCTTTTCATCGGCTCCAACTCCACTGACTCCTTCACCACGTCAGCTTCTGGATCGTCCCACTGATTTGGCAGTTGGACCTGATGGTCGCATATACGTCGTTGATTTCGGAA
ACAATTGCATCCGTGTCTTCTAG
```

Panel C

Conceptual translation of the NMD-sensitive region of the R-2 reporter. Asterisks represent STOP codons: in red, the STOP codon at the end of the coding region mCherry:linker:H2B that is recognized as a PTC by the NMD components; in green, the STOP codons in the NMDe (*i.e.*, arising from the out-of-frame last two exons of the *lin-41* sequence).

```
>conceptual translation_R-2 sequence

 M   A   I   I   K   E   F   M   R   F   K   V   H   M   E   G   S   V   N   G   H   E   F
ATG GCA ATT ATT AAA GAG TTT ATG CGT TTC AAG GTG CAT ATG GAG GGA TCT GTC AAT GGG CAT GAG TTT
 E   I   E   G   E   G   E   G   R   P   Y   E   G   T   Q   T   A   K   L   K   V   T   K
GAA ATT GAA GGT GAA GGA GAA GGC CGA CCA TAT GAG GGA ACA CAA ACC GCA AAA CTA AAG GTA ACT AAA

 G   G   P   L   P   F   A   W   D   I   L   S   P   Q   F   M   Y   G   S   K   A   Y   V
GGC GGA CCA TTA CCA TTC GCC TGG GAC ATC CTC TCT CCA CAG TTC ATG TAT GGA AGT AAA GCT TAT GTT

 K   H   P   A   D   I   P   D   Y   L   K   L   S   F   P   E   G   F   K   W   E   R   V
AAA CAT CCG GCA GAT ATA CCA GAT TAT TTG AAA CTT TCA TTC CCG GAG GGT TTT AAG TGG GAA CGC GTA

 M   N   F   E   D   G   G   V   V   T   V   T   Q   D   S   S   L   Q   D   G   E   F   I
ATG AAT TTT GAA GAC GGA GGA GTT GTT ACA GTG ACG CAA GAC TCA AGC CTC CAA GAT GGA GAA TTT ATT

 Y   K   V   K   L   R   G   T   N   F   P   S   D   G   P   V   M   Q   K   K   T   M   G
TAT AAA GTC AAA CTT CGA GGA ACG AAT TTC CCC TCG GAT GGA CCT GTT ATG CAG AAG AAG ACT ATG GGA

 W   E   A   S   S   E   R   M   Y   P   E   D   G   A   L   K   G   E   I   K   Q   R   L
TGG GAA GCT TCA AGT GAA AGA ATG TAC CCT GAA GAC GGT GCT CTT AAG GGA GAG ATT AAA CAA CGT CTT

 K   L   K   D   G   G   H   Y   D   A   E   V   K   T   T   Y   K   A   K   K   P   V   Q
AAA TTG AAA GAT GGA GGA CAT TAC GAT GCT GAG GTG AAG ACA ACT TAC AAA GCC AAA AAA CCA GTT CAG

 L   P   G   A   Y   N   V   N   I   K   L   D   I   T   S   H   N   E   D   Y   T   I   V
CTG CCA GGA GCG TAC AAT GTT AAT ATT AAA CTG GAT ATC ACC TCC CAC AAC GAG GAT TAC ACT ATC GTT

 E   Q   Y   E   R   A   E   G   R   H   S   T   G   G   M   D   E   L   Y   K   C   P   G
GAG CAA TAT GAA AGA GCT GAA GGG CGG CAC TCG ACA GGT GGC ATG GAT GAA TTG TAT AAG TGC CCG GGG

 D   R   W   S   S   T   G   G   G   R   S   R   T   S   P   P   K   P   S   A   K   G   A
GAT CGG TGG AGC TCC ACC GGT GGC GGC CGC TCT AGA ACT AGT CCA CCA AAG CCA TCT GCC AAG GGA GCC

 K   K   A   A   K   T   V   V   A   K   P   K   D   G   K   K   R   R   H   A   R   K   E
AAG AAG GCC GCC AAG ACC GTC GTT GCC AAG CCA AAG GAC GGA AAG AAG AGA CGT CAT GCC CGC AAG GAA

 S   Y   S   V   Y   I   Y   R   V   L   K   Q   V   H   P   D   T   G   V   S   S   K   A
TCG TAC TCC GTC TAC ATC TAC CGT GTT CTC AAG CAA GTT CAC CCA GAC ACC GGA GTC TCC TCC AAG GCC

 M   S   I   M   N   S   F   V   N   D   V   F   E   R   I   A   S   E   A   S   R   L   A
ATG TCT ATC ATG AAC TCC TTC GTC AAC GAT GTA TTC GAA CGC ATC GCT TCG GAA GCT TCC CGT CTT GCT

 H   Y   N   K   R   S   T   I   S   S   R   E   I   Q   T   A   V   R   L   I   L   P   G
CAT TAC AAC AAA CGC TCA ACG ATC TCA TCC CGC GAA ATT CAA ACC GCT GTC CGT TTG ATT CTC CCA GGA

 E   L   A   K   H   A   V   S   E   G   T   K   A   V   T   K   Y   T   S   S   K   *   L
GAA CTT GCC AAG CAC GCC GTG TCT GAG GGA ACC AAG GCC GTC ACC AAG TAC ACT TCC AGC AAG TAA CTT

 Q   Q   S   P   T   R   S   P   I   A   Q   K   H   V   R   D   E   G   L   R   K   *   R
CAA CAA TCA CCG ACT CGC AGT CCT ATC GCC CAG AAA CAT GTC AGA GAT GAA GGT TTA CGG AAG TGA AGG

 R   W   *   W   N   V   R   S   S   T   R   S   C   D   *   S   R   G   T   Y   T   C   M
AGA TGG TGA TGG AAT GTT CGT TCG TCC ACA AGG AGT TGT GAT TGA TCC AGA GGG ACA TAT ACT TGT ATG

 *   L   S   E   Q   S   S   S   S   L   C   V   *   *   Y   E   I   H   W   L   I   W   T
TGA CTC TCG GAA CAA TCG AGT TCA AGT CTT TGC GTC TGA TGA TAT GAG ATT CAT TGG CTC ATT TGG ACT

 W   T   R   P   Q   F   W   V   P   N   A   T   R   A   T   G   S   V   F   V   S   R   W
TGG ACC CGT CCC CAA TTC TGG GTT CCA AAT GCC ACA AGA GCT ACC GGC TCC GTA TTC GTC TCT CGG TGG
```

```
 S   I   R   C   S   S   L   F   I   G   S   N   S   T   D   S   F   T   T   S   A   S   G
TCC ATT CGG TGC TCC AGC CTT TTC ATC GGC TCC AAC TCC ACT GAC TCC TTC ACC ACG TCA GCT TCT GGA

 S   S   H   *   F   G   S   W   T   *   W   S   H   I   R   R   *   F   R   K   Q   L   H
TCG TCC CAC TGA TTT GGC AGT TGG ACC TGA TGG TCG CAT ATA CGT CGT TGA TTT CGG AAA CAA TTG CAT

 P   C   L   L
CCG TGT CTT CTA G
```
